# Inferring positive selection in humans from genomic data

**DOI:** 10.1186/s13323-015-0023-1

**Published:** 2015-04-01

**Authors:** Andreas Wollstein, Wolfgang Stephan

**Affiliations:** Section of Evolutionary Biology, Department of Biology II, University of Munich, Großhaderner Str. 2, 82152 Planegg-Martinsried, Germany

**Keywords:** Selective sweep, Evolution, Polygenic adaptation

## Abstract

Adaptation can be described as an evolutionary process that leads to an adjustment of the phenotypes of a population to their environment. In the classical view, new mutations can introduce novel phenotypic features into a population that leave footprints in the genome after fixation, such as selective sweeps. Alternatively, existing genetic variants may become beneficial after an environmental change and increase in frequency. Although they may not reach fixation, they may cause a shift of the optimum of a phenotypic trait controlled by multiple loci. With the availability of polymorphism data from various organisms, including humans and chimpanzees, it has become possible to detect molecular evidence of adaptation and to estimate the strength and target of positive selection. In this review, we discuss the two competing models of adaptation and suitable approaches for detecting the footprints of positive selection on the molecular level.

## Review

### Introduction

Understanding the genetic architecture and evolution of phenotypes that are present in populations adapting to heterogeneous environments has been a long-standing interest in evolutionary biology [1-3]. This question has been studied by means of quantitative genetics and population genetics. Quantitative genetics provides the methods to describe differences in the distribution of phenotypes, determine their heritability and map relevant regions controlling the phenotype in the genome [4]. In contrast, population genetics provides a framework to describe changes of allele frequencies that are known to be mostly determined by genetic drift [5] and selection [6]. The latter field produced a profound theory about the process of adaptation on the sequence level [7], which gave rise to an abundance of population genetic tools that can be applied to genetic data independent of phenotypes [8-14].

In the early years of the study of molecular adaptation, candidate genes with regard to certain phenotypes were conducted [12]. The progress in sequencing and genotyping methods, however, allowed researchers to produce genome-wide single nucleotide polymorphism data in humans and various other organisms [15-20]. This has motivated many genome-wide scans to search for signatures of positive selection [21-30] and quantify fitness effects of various classes of segregating sites [31-34].

Results from genome-wide scans, however, were often inconclusive [8,35,36]. The lack of reproducibility has been attributed to an insufficient power of the tests [37], the presence of masking signals of positive selection by purifying selection (for example, [38]) or complex demographic histories (for example, [39]). Furthermore, the classical model of adaptation in which single new mutations with large effects are favoured by recent positive selection has been questioned [40,41]. And the problem was raised whether evidence for more general models of adaptation (in particular those involving quantitative genetic variation) could be detected on the genomic level [39,40]. This latter issue became particularly interesting in the face of an influx of huge amounts of data from genome-wide association studies [42,43].

In this review, we summarize the population genetic and quantitative genetic models of adaptation and describe the methods to detect the footprints of adaptation in the genome. Furthermore, we provide examples of adaptation in humans that illustrate these theoretical accomplishments.

### Population genetic models of adaptation

Genetic adaptation is the result of fitness differences of alleles. Consider the alleles *a* and *A* at a bi-allelic locus in diploid organisms as mutant and wild type, respectively. A fitness value may be assigned to each possible genotype *aa*, *aA* and *AA*. Mutations are neutral if the fitness effects are equal (that is, *w*_*aa*_ = *w*_*aA*_ = *w*_*AA*_), which is the case for most of the genetic variation observed in humans [44]. In the classical model, positive selection occurs when the derived allele has a higher fitness than the ancestral allele, and negative (or purifying) selection, when the derived allele is detrimental to the organism. Balancing selection occurs in the case of heterozygote advantage and in situations of spatial and temporal heterogeneity of selection. Nucleotide changes in the DNA sequence may have some direct or indirect effect on the phenotype of the individual that generates a fitness advantage or disadvantage and hence are assumed to occur in coding regions of genes or regulatory sequences [45].

*Expected patterns of positive selection in the genome:* A beneficial mutation may rise quickly under positive selection. If the beneficial allele is going to fixation, genetic hitchhiking [46,47] results in depletion of variation around the selected site, also termed as selective sweep [46,47]. If the beneficial allele has not yet reached fixation, a sweep is called incomplete, partial, or ongoing. Sweep patterns that arise from a newly introduced mutation or migrant are considered as a ‘hard sweep’. If, however, the beneficial allele arises from standing variation, that is, after an environment change, the pattern of nucleotide polymorphism generated after fixation of the beneficial allele is called ‘soft sweep’ [48-50]. In this latter model, adaptation is not limited to the occurrence of new mutations and can therefore occur more rapidly after an environmental change [49]. The resulting pattern of variation of a soft sweep becomes very similar to that of a hard sweep in case the initial frequency of the beneficial allele is low. This situation may occur if the allele is initially in a mutation-selection balance and becomes positively selected after an environmental shift [46,47].

The genomic signatures of recent adaptation can be measured by means of the site frequency spectrum (SFS), which summarizes the counts of derived variants in a region. Under the action of positive directional selection, the SFS exhibits an excess of both rare and high-frequency derived variants around the selected site that are present in the population at the time of fixation of the beneficial allele [51,52]. The size of the region with depleted variation is expected to be larger when recombination is low and/or selection is strong [47,53], if hitchhiking has started from a selected allele with low frequency. The transient phase, until the beneficial mutation reaches fixation in the population, is inversely proportional to the population size [54].

Furthermore, in a subdivided population a frequency shift of a beneficial allele may lead to increased genetic differentiation between subpopulations in comparison to a population that has not been subjected to selection [55]. In its extreme, fixed differences between subpopulations may be observed.

The signature of linkage disequilibrium (LD) around the selected site is another characteristic of the hitchhiking process. LD emerges between pairs of sites due to non-random association of alleles. When selection is strong and a sweep is in progress, LD among hitchhiking alleles will strongly increase [56,57], due to limited time for recombination events to occur. However, after the beneficial allele driving hitchhiking has reached an intermediate frequency around 50%, LD between variants across the selected site decreases rapidly and eventually disappears when fixation has occurred. In contrast, the LD between polymorphisms on either side remains high and decreases only slowly. The establishment of the well-known long-range haplotypes in a population [21] is a consequence of the strong LD around the selected site in the first half of the selective phase (until the beneficial allele reaches intermediate frequency). Therefore, these extended haplotypes can be used to detect incomplete (ongoing) sweeps that are typical for humans [9,36]. The use of LD has the advantage that it is relatively robust against purifying selection [38].

Once a beneficial mutation has been fixed in a population the signature of linkage disequilibrium decreases and the pattern of polymorphism in the neighbourhood can be restored. The time range to detect these LD signatures of recent adaptation in a single population is rather limited (for example, in the scale of 10,000 years in the case of humans [36]) and measurable only when adaptation is still ongoing or has only recently ceased. The fixed differences between populations or species remain evident much longer (millions of years, humans compared to chimp, [36]).

These latter genomic signatures of positive selection, however, may not be unique. It is known that population expansion as well as sudden decreases in population size (bottlenecks) can result in similar genomic patterns, such as an excess of rare and intermediate-frequency derived variants, respectively [58,59]. For example, while human populations were migrating out of Africa, consecutive population bottlenecks followed by population expansion occurred [60,61]. Such a cascade of demographic events is expected to leave patterns in the genome that are very similar to the one of selective sweeps [62].

Furthermore, population structure can mimic the signature of balancing selection [63]. The inference of the demographic history of a population can in addition be confounded by the genotyping technology leading to single nucleotide polymorphism (SNP) ascertainment bias [64,65]. Choosing SNPs from a too small discovery sample for genotyping can skew the resulting site frequency spectrum toward intermediate frequencies.

Alternative modes of selection may also result in similar signatures as those produced by positive directional selection. In particular, background selection may also lead to a depletion of variation [66,67], yet without causing shifts of low- and high-frequency-derived variants in the SFS. This signature may resemble that of multiple selective sweeps (recurrent sweeps; [68]) and may result in a lack of high-frequency-derived variants [69]. Selective sweeps may also be difficult to distinguish from recombination hotspots [70]. If recombination is strong, the region of depleted variation may become too small to be recognized. In contrast, a recombination cold spot can generate a pattern of increased LD that is similar as the pattern of a sweep in progress [71]. Furthermore, varying recombination rate on a fine scale may also confound the long-range haplotype signature of sweeps.

A common statistical approach dealing with these difficulties is to derive a likelihood by comparing a statistical null model that includes all the aforementioned non-selective effects to an alternative model that in addition contains positive selection. Many of the confounding factors, however, are difficult to model jointly in a likelihood framework. In an alternative approach, summary statistics are constructed that quantify specific patterns of selective forces and are applied genome-wide. Regions with the strongest signals are considered as outliers. Statistical significance is then assessed by simulating a null model using the coalescent [72]. In the following, we review statistical approaches and their applications taking these confounding effects into account.

*Statistical tests to detect deviations from neutrality:* Several tests have been developed that make use of the aforementioned signatures of hitchhiking, that is, the reduction of genetic variation, the skew in the frequency spectrum and the pattern of linkage disequilibrium. These tests may be broadly categorized into three classes: (i) tests that use only data from one population, (ii) tests that compare genetic signatures among multiple populations and (iii) comparative tests that use a closely related species as an out-group. The tests can be further classified into model-free and model-based methods. The latter use the neutral theory [5] to build the null hypothesis and can be applied to compare single candidate regions to a neutral expectation, when full genome data is not available. In contrast, model-free methods try to quantify the characteristic signatures of hitchhiking and are usually applied in an outlier approach to genome-wide data. Regions that show the strongest signals are assumed to be candidates for sweeps [8,10,73].

The most widely used method in the first class of tests is Tajima’s D statistic [74] that compares the number of segregating sites to an expected value when the population size is assumed to be constant over time (standard neutral model). Large positive values indicate an excess of variation in the tested region that could be due to balancing selection, whereas negative values indicate a depletion of variation due to positive directional selection. The interpretation of the Tajima’s D statistic, however, may be ambiguous as the demographic history of a population needs to be taken into account. Therefore, several more recent developments corrected Tajima’s D statistic, for instance, by including population size changes [75] or SNP ascertainment bias [76] that can arise from genotyping technology [64].

The Fay and Wu’s H test [52] uses, in addition, data from an out-group species to get information of the ancestral state of a polymorphism and detect selective sweeps by an excess of high-frequency-derived polymorphisms. In contrast, the Fu and Li’s D statistic [77] takes advantage of low-frequency variation that is enriched in regions that recently underwent genetic hitchhiking. The maximum frequency of derived mutations (MFDM) test [78] utilizes the MFDM to estimate the presence of an unbalanced tree topology in a given sample that is thought to arise in the adjacency of a locus that is under positive selection due to hitchhiking [46,52]. In line with coalescent theory, the tree topology is independent of changes in population size, which makes the MFDM statistics evidently robust against demographic events, such as bottlenecks or expansions [78]. To obtain good estimates for the MFDM statistics, large sample sizes of at least 42 chromosomes (21 diploids) are necessary [78] that have to be unaffected by migration, admixture or any hidden population substructure.

A statistic that uses the full site frequency spectrum has been introduced by Kim and Stephan [54]. Here, a composite likelihood ratio (CLR) is calculated by multiplying the probabilities of all polymorphic sites of a genomic region, which makes it possible to estimate the strength and location of a selective sweep. The method returns a likelihood of a complete sweep compared to a population that evolves under standard neutrality, and an estimate about the selection parameter and the target of selection. This test has been further developed by Nielsen *et al*. [8] to detect deviations from a background spectrum that includes deviations from neutrality due to demographic history and SNP ascertainment bias under the assumption that the selective sweep has been completed. A demographic model consisting of two epochs of population sizes has been incorporated into the CLR approach by Williamson *et al*. [31]. Finally, LD has been combined with this composite likelihood framework by Pavlidis *et al*. [79], which is reducing the number of false positives. Currently, the most advanced CLR-based test is SweeD [80] that includes a demographic model with an arbitrary number of instantaneous changes in population size [81]. The power of this test increases with up to a sample size of about 500.

A large fraction of model-free tests are also based on the patterns of LD. Many tests take advantage of the haplotype homozygosity as introduced by Sabeti *et al*. [21], which is a measure of genetic diversity regarding multiple polymorphic sites [82]. The decay of the extended haplotype homozygosity (EHH) as calculated step by step from a defined core haplotype was designed as a test for positive selection. This test, however, cannot easily distinguish between complete and incomplete sweeps. Several modifications of the EHH test statistic have been introduced that account for the confounding effect of varying recombination rates. The relative extended haplotype homozygosity (REHH) is defined to be the extended homozygosity of a core haplotype divided by the homozygosity of the remaining core haplotypes combined [83]. The integrated haplotype score (iHS) as proposed by Voight *et al*. [22] compares the decay of the ancestral allele against the derived allele. If the derived allele is beneficial, its underlying haplotype will take longer to decay than the ancestral one. While this test cannot be applied to sites that are already fixed, it is useful to detect recent sweeps that are still in progress (that is, incomplete sweeps). As the latter mentioned tests do not compare the observation with a theoretical expectation, they are mostly used in a statistical outlier approach.

The second class of tests compares recently diverging populations under the assumption that adaptation was acting differently on the populations. A test for detecting differentiation in allele frequencies between populations by means of Wright’s fixation index *F*_ST_ [84] has been first formulated by Lewontin and Krakauer [85]. This idea has been incorporated into various frequency- and LD-based test statistics. The CLR approach has been extended by Chen *et al*. [86]. It models population structure by multi-locus allele frequency differentiation between two populations (XP-CLR). However, population size changes and associations between polymorphic sites were not considered in the model. The model-free Rsb measure proposed by Tang *et al*. [29] compares the haplotype homozygosity decay at homologous sites between two populations that diverged recently. Similarly, the XP-EHH method [83] compares the homozygosity decay among different populations. The latter tests take advantage of the assumption that local adaptation increases population differentiation compared to neutrally evolving subpopulations. Another extension of measuring population differentiation between populations on a haplotype level is a method proposed by Fariello *et al*. [87] and Ferrer-Admetlla *et al*. [88] that has been shown to have more power to detect soft sweeps over SFS-based methods [88]. A combination of class one and class two tests has been proposed in [89]. The composite of multiple signals (CMS) test combines the different priors of detecting extended haplotypes (XP-EHH, iHS), high-frequency-derived alleles (iHS), and polymorphic sites that exhibit population differentiation and results in a score that represents a posterior probability that a certain variant is under selection [89].

The third class of tests uses the information of an out-group species to detect selection. Most widely used is the dN/dS ratio, also known as Ka/Ks statistic [90]. The basic idea is that the ratio of non-synonymous and synonymous substitutions is close to one under neutrality. The Hudson-Kreitmann-Aquadé test (HKA, [91]) compares polymorphisms within species by means of Watterson’s estimator [92] and divergence between species across two or more loci. Under neutrality, they are expected to be identical, which is tested by means of a goodness of fit test. In contrast, the McDonald-Kreitman test compares polymorphism within populations and divergence between species at single loci for two classes of sites (for example, synonymous and non-synonymous sites) [93].

### Quantitative genetic models of adaptation

Quantitative genetic models of adaptation date back to the time before the genetic mechanisms of inheritance were fully discovered [1,94]. Quantitative phenotypes in a population are characterized by a distribution of gradual differences among individuals that are controlled by a multitude of genes. In varying environments, different phenotypes may be favoured. This leads to a change in the population mean phenotype that is known to depend on the additive genetic variation present in the population. When a population deviates from its optimum, mutations are favoured according to their effect size and distance to the optimum. The mean step size of such an adaptive walk has been shown to be approximately exponentially distributed [1]; that is, alleles with larger effects are favoured when the population resides far from the optimum, whereas alleles with smaller effects are favoured during the adaptive fine-tuning close to the population optimum.

The impact of beneficial mutations in the process of adaptation depends on the mutation rate and population size [95]. In humans, the most non-synonymous mutations have been shown to be neutral (27% to 29% [33]) or mildly deleterious (30% to 42% [31,33]). In comparison with chimpanzees, 10% to 20% of the fixations appear to be adaptive [33]. However, beneficial mutations that lead to fixation in recent time have been shown to be rare (1% [96]), so that adaptation from standing variation may be the most important mode of recent adaptation.

In this scenario, classical selective sweeps play only a role if the beneficial alleles are driven to fixation from low frequency by strong selection [40,97]. Instead, small frequency shifts of selected alleles at the quantitative trait loci driving a trait value towards its optimum may occur predominantly.

In case the trait optima of populations are ordered along clines [98,99], effective alleles are expected to change in frequency accordingly [40]. This may be detected by means of the Lewontin and Krakauer test [85] and other *F*_ST_-based statistics (for example, [100]). To be able to distinguish these adaptive frequency changes from drift, Coop *et al*. [101] proposed a model that analyses whether allele frequencies correlate with environmental variables along a population gradient. A test for polygenic adaptation that also incorporates estimates of phenotypic values from genome-wide association data and compares those with environmental variables has been recently introduced by Berg and Coop [102]. However, phenotypic and genotypic data for many populations are required for this test.

### Evidence for adaptation in humans

As the migration out of Africa [103] and the settlement around the world exposed humans to different environmental conditions with regard to temperature, amount of light, humidity, oxygen levels, and agriculture [104], many adaptations in non-African populations must have occurred in the recent past [105]. In line with this, positive selection has been shown to be a less important determinant in various African populations [106]. The most accepted examples from different genome scans show human adaptations to (i) agriculture [104], (ii) environmental variables, such as amount of light, temperature, or oxygen levels, and (iii) pathogen resistance [107-109].

The most prominent example of adaptation in humans to agriculture is the ability to digest lactose from milk products in adulthood [110]. Indeed, an extended haplotype homozygosity as a signature for a selective sweep around the LCT gene was observed [22,83]. The activity of the LCT gene is usually reduced in adult mammals [110]. However, the presence of the beneficial mutation provides a selective advantage of about 1.4% to 19% [111]. The most likely explanation for the evolutionary advantage of the mutation is the additional caloric and calcium source it produces because it reduces the risk for diseases related to bone mineralization caused by a lack of vitamin D [110,112]. The frequency of the allele associated with lactase persistence has been shown to decline from Northwest Europe to the southern populations [110] and the mutation is absent in African populations. In African rural, populations show strong evidence for parallel adaptation to digest lactose from milk products. Other alleles have been associated with lactase persistence [113] that show similar LD patterns and high selection coefficients of 4% to 9% [113,114].

Skin pigmentation is another example of adaptation to environmental conditions in humans. It is known to be controlled by the amount of eumelanin and pheomelanin that are produced in the melanosomes [115,116]. The dark pigmented skin is assumed to be ancestral, whereas lighter pigmented skin has emerged after the migration out of Africa [117]. Skin colour has long been speculated to evolve under positive selection and is another example for convergent evolution [115,117,118]. Many genes have been shown to be associated with variation in skin colour in different human populations [115,116]. The *MC1R* gene is a main switch in the production of the lighter pheomelanin and darker eumelanin pigments in the melanosomes [116]. Strong selection for the persistence of the dark pigment has been found in African [119] and southern European populations [120]. The gene *SLC24A5* regulates calcium levels in melanosomes and has been associated with lighter pigmentation in Europeans [121]. In genome-wide scans, it has been shown that *SLC24A5* is surrounded by a region of decreased variability and increased LD levels [22,23,83,117] and is substantially differentiated among different populations [23,105,122]. In East Asian populations, another candidate gene, *OCA2*, has been shown to be subject to positive selection [118]. Furthermore, there are several other candidate genes, such as *UGT1A* and *BNC2* that are associated with skin pigmentation [123]. However, an adaptive signature has not been observed for these genes yet, most likely due to lower effect sizes so that the establishment of a sweep signature and/or frequency changes become too small to be identified.

Human height is a classical quantitative trait that has been studied since the beginning of the last century [124-126] and shows evidence for phenotypic adaptation to different environmental factors, such as temperature (for example, Bergmans rule [40]), with extreme differences among populations of up to 30 cm [127]. More than 180 loci have been associated with it [128], with no evidence of selective sweeps so far. Turchin *et al*. [129] demonstrated that alleles that contribute to a tall stature are enriched in northern European populations, which is better explained by small selection coefficients of 0.001% to 0.1% than drift. Since human height can be expected to be under stabilizing selection [115,116], the probability of observing selective sweeps is rather low [97].

Another example of parallel adaptation to low oxygen levels in high altitude has been described in Tibetan, Andean, and Ethiopian populations. Tibetans and Ethiopians adapted differently to the low oxygen levels compared to Andeans [130]. Andeans show an increased haemoglobin blood concentration that elevates the oxygen transport in blood, whereas Tibetans and Ethiopians exhibit an increased lung capacity and breathing rate [130]. The *EPAS1* and *EGLN1* genes show strong signatures of selective sweeps in Tibetans; that is, an increased differentiation in allele frequency compared to East Asian populations and an increased LD [131-133]. Variants of the *EPAS1* and *EGLN1* genes have been associated with haemoglobin concentration levels in blood [134,135]. It has been shown that the *EPAS1* gene has likely been introgressed from an archaic human, the Denisovans into the Tibetans [136]. In the Andean population, different genes (*NOS2A* and *PRKAA1)* have been identified as targets of adaptation [131,137]. Ethiopian high-altitude populations that have a similar phenotype as Tibetan populations, also show a different set of genes (*CBARA1*, *VAV3*, *ARNT2* and *THRB)*, with evidence for positive selection [138]. Variants associated with haemoglobin variation in Tibetans do not overlap with variants associated in Ethiopians [139].

## Conclusions

Classical sweeps have been shown to be rare in humans [13,96,105] and, if they exist, they occur around loci with large-effects alleles. As selective sweeps are rare in humans (in contrast to species with large effective sizes such as Drosophila), the emphasis of human population genetics in the near future must be to identify adaptive signatures for polygenic phenotypes. There is an urgent need for more theoretical modelling and better statistical methods to analyse the evolution of polygenic traits for populations of varying environments and demographies.

## Abbreviations

CLR: composite likelihood ratio
CMS: composite of multiple signals
EHH: extended haplotype homozygosity
iHS: integrated haplotype score
LD: linkage disequilibrium
MFDM: maximum frequency of derived mutations
REHH: relative extended haplotype homozygosity
SFS: site frequency spectrum
SNP: single nucleotide polymorphism
XP-CLR: cross population composite likelihood ratio
XP-EHH: cross population extended haplotype homozygosity

## References

[1] Orr HA (2005). The genetic theory of adaptation: a brief history. Nat Rev Genet.

[2] Mackay TF (2001). The genetic architecture of quantitative traits. Annu Rev Genet.

[3] Fu W, O’Connor TD, Akey JM (2013). Genetic architecture of quantitative traits and complex diseases. Curr Opin Genet Dev.

[4] Hill WG (2010). Understanding and using quantitative genetic variation. Philos Trans R Soc Lond B Biol Sci.

[5] Kimura M (1983). The neutral theory of molecular evolution.

[6] Orr HA (2009). Fitness and its role in evolutionary genetics. Nat Rev Genet.

[7] Crow JF, Kimura M (1970). An introduction to population genetics theory.

[8] Nielsen R (2005). Molecular signatures of natural selection. Annu Rev Genet.

[9] Nielsen R, Hellmann I, Hubisz M, Bustamante C, Clark AG (2007). Recent and ongoing selection in the human genome. Nat Rev Genet.

[10] Pavlidis P, Hutter S, Stephan W (2008). A population genomic approach to map recent positive selection in model species. Mol Ecol.

[11] Stephan W (2010). Detecting strong positive selection in the genome. Mol Ecol Resour.

[12] Vitti JJ, Grossman SR, Sabeti PC (2013). Detecting natural selection in genomic data. Annu Rev Genet.

[13] Fu W, Akey JM (2013). Selection and adaptation in the human genome. Annu Rev Genomics Hum Genet.

[14] Jeong C, Di Rienzo A (2014). Adaptations to local environments in modern human populations. Curr Opin Genet Dev.

[15] International HapMap Consortium (2003). The International HapMap Project. Nature.

[16] Chimpanzee Sequencing and Analysis Consortium (2005). Initial sequence of the chimpanzee genome and comparison with the human genome. Nature.

[17] International HapMap Consortium (2005). A haplotype map of the human genome. Nature.

[18] Abecasis GR, Altshuler D, Auton A, Brooks LD, Durbin RM, 1000 Genomes Project Consortium (2010). A map of human genome variation from population-scale sequencing. Nature.

[19] Cann HM, de Toma C, Cazes L, Legrand M-F, Morel V, Piouffre L (2002). A human genome diversity cell line panel. Science.

[20] Hinds DA, Stuve LL, Nilsen GB, Halperin E, Eskin E, Ballinger DG (2005). Whole-genome patterns of common DNA variation in three human populations. Science.

[21] Sabeti PC, Reich DE, Higgins JM, Levine HZP, Richter DJ, Schaffner SF (2002). Detecting recent positive selection in the human genome from haplotype structure. Nature.

[22] Voight BF, Kudaravalli S, Wen X, Pritchard JK (2006). A map of recent positive selection in the human genome. PLoS Biol.

[23] Lao O, de Gruijter JM, van Duijn K, Navarro A, Kayser M (2007). Signatures of positive selection in genes associated with human skin pigmentation as revealed from analyses of single nucleotide polymorphisms. Ann Hum Genet.

[24] Carlson CS, Thomas DJ, Eberle MA, Swanson JE, Livingston RJ, Rieder MJ (2005). Genomic regions exhibiting positive selection identified from dense genotype data. Genome Res.

[25] Bustamante CD, Fledel-Alon A, Williamson S, Nielsen R, Hubisz MT, Glanowski S (2005). Natural selection on protein-coding genes in the human genome. Nature.

[26] Akey JM, Zhang G, Zhang K, Jin L, Shriver MD (2002). Interrogating a high-density SNP map for signatures of natural selection. Genome Res.

[27] Shriver MD, Kennedy GC, Parra EJ, Lawson HA, Sonpar V, Huang J (2004). The genomic distribution of population substructure in four populations using 8,525 autosomal SNPs. Hum Genomics.

[28] Kelley JL, Madeoy J, Calhoun JC, Swanson W, Akey JM (2006). Genomic signatures of positive selection in humans and the limits of outlier approaches. Genome Res.

[29] Tang K, Thornton KR, Stoneking M (2007). A new approach for using genome scans to detect recent positive selection in the human genome. PLoS Biol.

[30] Kimura R, Ohashi J, Matsumura Y, Nakazawa M, Inaoka T, Ohtsuka R (2008). Gene flow and natural selection in oceanic human populations inferred from genome-wide SNP typing. Mol Biol Evol.

[31] Williamson SH, Hernandez R, Fledel-Alon A, Zhu L, Nielsen R, Bustamante CD (2005). Simultaneous inference of selection and population growth from patterns of variation in the human genome. Proc Natl Acad Sci U S A.

[32] Eyre-Walker A, Keightley PD (1999). High genomic deleterious mutation rates in hominids. Nature.

[33] Boyko AR, Williamson SH, Indap AR, Degenhardt JD, Hernandez RD, Lohmueller KE (2008). Assessing the evolutionary impact of amino acid mutations in the human genome. PLoS Genet.

[34] Eyre-Walker A, Woolfit M, Phelps T (2006). The distribution of fitness effects of new deleterious amino acid mutations in humans. Genetics.

[35] Akey JM (2009). Constructing genomic maps of positive selection in humans: where do we go from here?. Genome Res.

[36] Sabeti PC, Schaffner SF, Fry B, Lohmueller J, Varilly P, Shamovsky O (2006). Positive natural selection in the human lineage. Science.

[37] Zhai W, Nielsen R, Slatkin M (2009). An investigation of the statistical power of neutrality tests based on comparative and population genetic data. Mol Biol Evol.

[38] Enard D, Messer PW, Petrov DA (2014). Genome-wide signals of positive selection in human evolution. Genome Res.

[39] Teshima KM, Coop G, Przeworski M (2006). How reliable are empirical genomic scans for selective sweeps?. Genome Res.

[40] Pritchard JK, Pickrell JK, Coop G (2010). The genetics of human adaptation: hard sweeps, soft sweeps, and polygenic adaptation. Curr Biol.

[41] Pritchard JK, Di Rienzo A (2010). Adaptation - not by sweeps alone. Nat Rev Genet.

[42] McCarthy MI, Abecasis GR, Cardon LR, Goldstein DB, Little J, Ioannidis JPA (2008). Genome-wide association studies for complex traits: consensus, uncertainty and challenges. Nat Rev Genet.

[43] Hindorff LA, Sethupathy P, Junkins HA, Ramos EM, Mehta JP, Collins FS (2009). Potential etiologic and functional implications of genome-wide association loci for human diseases and traits. Proc Natl Acad Sci U S A.

[44] Hellmann I, Ebersberger I, Ptak SE, Pääbo S, Przeworski M (2003). A neutral explanation for the correlation of diversity with recombination rates in humans. Am J Hum Genet.

[45] Zhen Y, Andolfatto P (2012). Methods to detect selection on noncoding DNA. Methods Mol Biol.

[46] Kaplan NL, Hudson RR, Langley CH (1989). The “hitchhiking effect” revisited. Genetics.

[47] Stephan W, Wiehe THE, Lenz MW (1992). The effect of strongly selected substitutions on neutral polymorphism: analytical results based on diffusion theory. Theor Popul Biol.

[48] Innan H, Kim Y (2004). Pattern of polymorphism after strong artificial selection in a domestication event. Proc Natl Acad Sci U S A.

[49] Hermisson J, Pennings PS (2005). Soft sweeps: molecular population genetics of adaptation from standing genetic variation. Genetics.

[50] Przeworski M, Coop G, Wall JD (2005). The signature of positive selection on standing genetic variation. Evolution.

[51] Braverman JM, Hudson RR, Kaplan NL, Langley CH, Stephan W (1995). The hitchhiking effect on the site frequency spectrum of DNA polymorphisms. Genetics.

[52] Fay JC, Wu CI (2000). Hitchhiking under positive Darwinian selection. Genetics.

[53] Maynard Smith J, Haigh J (1974). The hitch-hiking effect of a favourable gene. Genet Res.

[54] Kim Y, Stephan W (2002). Detecting a local signature of genetic hitchhiking along a recombining chromosome. Genetics.

[55] Kim Y (2013). Stochastic patterns of polymorphism after a selective sweep over a subdivided population. Genet Res.

[56] Kim Y, Nielsen R (2004). Linkage disequilibrium as a signature of selective sweeps. Genetics.

[57] Stephan W, Song YS, Langley CH (2006). The hitchhiking effect on linkage disequilibrium between linked neutral loci. Genetics.

[58] Tajima F (1989). The effect of change in population size on DNA polymorphism. Genetics.

[59] Evans SN, Shvets Y, Slatkin M (2007). Non-equilibrium theory of the allele frequency spectrum. Theor Popul Biol.

[60] DeGiorgio M, Jakobsson M, Rosenberg NA (2009). Out of Africa: modern human origins special feature: explaining worldwide patterns of human genetic variation using a coalescent-based serial founder model of migration outward from Africa. Proc Natl Acad Sci U S A.

[61] Veeramah KR, Hammer MF (2014). The impact of whole-genome sequencing on the reconstruction of human population history. Nat Rev Genet.

[62] Sousa V, Peischl S, Excoffier L (2014). Impact of range expansions on current human genomic diversity. Curr Opin Genet Dev.

[63] Muirhead CA (2001). Consequences of population structure on genes under balancing selection. Evolution.

[64] Clark AG, Hubisz MJ, Bustamante CD, Williamson SH, Nielsen R (2005). Ascertainment bias in studies of human genome-wide polymorphism. Genome Res.

[65] Nielsen R (2004). Population genetic analysis of ascertained SNP data. Hum Genomics.

[66] Stephan W (2010). Genetic hitchhiking versus background selection: the controversy and its implications. Philos Trans R Soc Lond B Biol Sci.

[67] Charlesworth B, Morgan MT, Charlesworth D (1993). The effect of deleterious mutations on neutral molecular variation. Genetics.

[68] Stephan W (1995). An improved method for estimating the rate of fixation of favorable mutations based on DNA polymorphism data. Mol Biol Evol.

[69] Kim Y (2006). Allele frequency distribution under recurrent selective sweeps. Genetics.

[70] Reed FA, Tishkoff SA (2006). Positive selection can create false hotspots of recombination. Genetics.

[71] McVean G (2007). The structure of linkage disequilibrium around a selective sweep. Genetics.

[72] Hudson RR (1990). Others: gene genealogies and the coalescent process. Oxford Surveys Evol Biol.

[73] Scheinfeldt LB, Tishkoff SA (2013). Recent human adaptation: genomic approaches, interpretation and insights. Nat Rev Genet.

[74] Tajima F (1989). Statistical method for testing the neutral mutation hypothesis by DNA polymorphism. Genetics.

[75] Zivković D, Wiehe T (2008). Second-order moments of segregating sites under variable population size. Genetics.

[76] Ramírez-Soriano A, Nielsen R (2009). Correcting estimators of theta and Tajima’s D for ascertainment biases caused by the single-nucleotide polymorphism discovery process. Genetics.

[77] Fu YX, Li WH (1993). Statistical tests of neutrality of mutations. Genetics.

[78] Li H (2011). A new test for detecting recent positive selection that is free from the confounding impacts of demography. Mol Biol Evol.

[79] Pavlidis P, Jensen JD, Stephan W (2010). Searching for footprints of positive selection in whole-genome SNP data from nonequilibrium populations. Genetics.

[80] Pavlidis P, Živkovic D, Stamatakis A, Alachiotis N (2013). SweeD: likelihood-based detection of selective sweeps in thousands of genomes. Mol Biol Evol.

[81] Živković D, Stephan W (2011). Analytical results on the neutral non-equilibrium allele frequency spectrum based on diffusion theory. Theor Popul Biol.

[82] Sabatti C, Risch N (2002). Homozygosity and linkage disequilibrium. Genetics.

[83] Sabeti PC, Varilly P, Fry B, Lohmueller J, Hostetter E, Cotsapas C (2007). Genome-wide detection and characterization of positive selection in human populations. Nature.

[84] Wright S (1950). Genetical structure of populations. Nature.

[85] Lewontin RC, Krakauer J (1973). Distribution of gene frequency as a test of the theory of the selective neutrality of polymorphisms. Genetics.

[86] Chen H, Patterson N, Reich D (2010). Population differentiation as a test for selective sweeps. Genome Res.

[87] Fariello MI, Boitard S, Naya H, SanCristobal M, Servin B (2013). Detecting signatures of selection through haplotype differentiation among hierarchically structured populations. Genetics.

[88] Ferrer-Admetlla A, Liang M, Korneliussen T, Nielsen R (2014). On detecting incomplete soft or hard selective sweeps using haplotype structure. Mol Biol Evol.

[89] Grossman SR, Shlyakhter I, Shylakhter I, Karlsson EK, Byrne EH, Morales S (2010). A composite of multiple signals distinguishes causal variants in regions of positive selection. Science.

[90] Miyata T, Yasunaga T (1980). Molecular evolution of mRNA: a method for estimating evolutionary rates of synonymous and amino acid substitutions from homologous nucleotide sequences and its application. J Mol Evol.

[91] Hudson RR, Kreitman M, Aguadé M (1987). A test of neutral molecular evolution based on nucleotide data. Genetics.

[92] Watterson GA (1975). On the number of segregating sites in genetical models without recombination. Theor Popul Biol.

[93] McDonald JH, Kreitman M (1991). Adaptive protein evolution at the Adh locus in Drosophila. Nature.

[94] Barton NH (1998). The geometry of adaptation. Nature.

[95] Elena SF, Wilke CO, Ofria C, Lenski RE (2007). Effects of population size and mutation rate on the evolution of mutational robustness. Evolution.

[96] Hernandez RD, Kelley JL, Elyashiv E, Melton SC, Auton A, McVean G (2011). Classic selective sweeps were rare in recent human evolution. Science.

[97] Wollstein A, Stephan W (2014). Adaptive fixation in two-locus models of stabilizing selection and genetic drift. Genetics.

[98] Haldane JBS (1948). The theory of a cline. J Genet.

[99] Barton NH (1979). Gene flow past a cline. Heredity.

[100] Rosenberg NA, Li LM, Ward R, Pritchard JK (2003). Informativeness of genetic markers for inference of ancestry. Am J Hum Genet.

[101] Coop G, Witonsky D, Di Rienzo A, Pritchard JK (2010). Using environmental correlations to identify loci underlying local adaptation. Genetics.

[102] Berg JJ, Coop G (2014). A population genetic signal of polygenic adaptation. PLoS Genet.

[103] Stoneking M, Krause J (2011). Learning about human population history from ancient and modern genomes. Nat Rev Genet.

[104] Beja-Pereira A, Caramelli D, Lalueza-Fox C, Vernesi C, Ferrand N, Casoli A (2006). The origin of European cattle: evidence from modern and ancient DNA. Proc Natl Acad Sci U S A.

[105] Coop G, Pickrell JK, Novembre J, Kudaravalli S, Li J, Absher D (2009). The role of geography in human adaptation. PLoS Genet.

[106] Granka JM, Henn BM, Gignoux CR, Kidd JM, Bustamante CD, Feldman MW (2012). Limited evidence for classic selective sweeps in African populations. Genetics.

[107] Daub JT, Hofer T, Cutivet E, Dupanloup I, Quintana-Murci L, Robinson-Rechavi M (2013). Evidence for polygenic adaptation to pathogens in the human genome. Mol Biol Evol.

[108] Siddle KJ, Quintana-Murci L (2014). The Red Queen’s long race: human adaptation to pathogen pressure. Curr Opin Genet Dev.

[109] Barreiro LB, Quintana-Murci L (2010). From evolutionary genetics to human immunology: how selection shapes host defence genes. Nat Rev Genet.

[110] Swallow DM (2003). Genetics of lactase persistence and lactose intolerance. Annu Rev Genet.

[111] Bersaglieri T, Sabeti PC, Patterson N, Vanderploeg T, Schaffner SF, Drake JA (2004). Genetic signatures of strong recent positive selection at the lactase gene. Am J Hum Genet.

[112] Gerbault P, Liebert A, Itan Y, Powell A, Currat M, Burger J (2011). Evolution of lactase persistence: an example of human niche construction. Philos Trans R Soc Lond B Biol Sci.

[113] Tishkoff SA, Reed FA, Ranciaro A, Voight BF, Babbitt CC, Silverman JS (2007). Convergent adaptation of human lactase persistence in Africa and Europe. Nat Genet.

[114] Breton G, Schlebusch CM, Lombard M, Sjödin P, Soodyall H, Jakobsson M (2014). Lactase persistence alleles reveal partial East African ancestry of Southern African Khoe pastoralists. Curr Biol.

[115] Sturm RA (2009). Molecular genetics of human pigmentation diversity. Hum Mol Genet.

[116] Liu F, Wen B, Kayser M (2013). Colorful DNA polymorphisms in humans. Semin Cell Dev Biol.

[117] Norton HL, Kittles RA, Parra E, McKeigue P, Mao X, Cheng K (2007). Genetic evidence for the convergent evolution of light skin in Europeans and East Asians. Mol Biol Evol.

[118] Sturm RA, Duffy DL (2012). Human pigmentation genes under environmental selection. Genome Biol.

[119] Harding RM, Healy E, Ray AJ, Ellis NS, Flanagan N, Todd C (2000). Evidence for variable selective pressures at MC1R. Am J Hum Genet.

[120] Martínez-Cadenas C, López S, Ribas G, Flores C, García O, Sevilla A (2013). Simultaneous purifying selection on the ancestral MC1R allele and positive selection on the melanoma-risk allele V60L in south Europeans. Mol Biol Evol.

[121] Lamason RL, Mohideen M-APK, Mest JR, Wong AC, Norton HL, Aros MC (2005). SLC24A5, a putative cation exchanger, affects pigmentation in zebrafish and humans. Science.

[122] Pickrell JK, Coop G, Novembre J, Kudaravalli S, Li JZ, Absher D (2009). Signals of recent positive selection in a worldwide sample of human populations. Genome Res.

[123] Jacobs LC, Wollstein A, Lao O, Hofman A, Klaver CC, Uitterlinden AG (2013). Comprehensive candidate gene study highlights UGT1A and BNC2 as new genes determining continuous skin color variation in Europeans. Hum Genet.

[124] Galton F (1886). Hereditary stature. Nature.

[125] Blakeslee AF (1914). Corn and men. The interacting influence of heredity and environment - movements for betterment of men, or corn, or any other living thing, one-sided unless they take both fact. J Hered.

[126] Fisher RA (1919). The correlation between relatives on the supposition of Mendelian inheritance. Trans R Soc Edinb.

[127] Mendizabal I, Marigorta UM, Lao O, Comas D (2012). Adaptive evolution of loci covarying with the human African Pygmy phenotype. Hum Genet.

[128] Lango Allen H, Estrada K, Lettre G, Berndt SI, Weedon MN, Rivadeneira F (2010). Hundreds of variants clustered in genomic loci and biological pathways affect human height. Nature.

[129] Turchin MC, Chiang CWK, Palmer CD, Sankararaman S, Reich D, Genetic Investigation of Anthropometric Traits (GIANT) Consortium (2012). Evidence of widespread selection on standing variation in Europe at height-associated SNPs. Nat Genet.

[130] Beall CM (2007). Two routes to functional adaptation: Tibetan and Andean high-altitude natives. Proc Natl Acad Sci U S A.

[131] Bigham A, Bauchet M, Pinto D, Mao X, Akey JM, Mei R (2010). Identifying signatures of natural selection in Tibetan and Andean populations using dense genome scan data. PLoS Genet.

[132] Bigham AW, Wilson MJ, Julian CG, Kiyamu M, Vargas E, Leon-Velarde F (2013). Andean and Tibetan patterns of adaptation to high altitude. Am J Hum Biol.

[133] Yi X, Liang Y, Huerta-Sanchez E, Jin X, Cuo ZXP, Pool JE (2010). Sequencing of 50 human exomes reveals adaptation to high altitude. Science.

[134] Beall CM, Cavalleri GL, Deng L, Elston RC, Gao Y, Knight J (2010). Natural selection on EPAS1 (HIF2α) associated with low hemoglobin concentration in Tibetan highlanders. Proc Natl Acad Sci U S A.

[135] Simonson TS, Yang Y, Huff CD, Yun H, Qin G, Witherspoon DJ (2010). Genetic evidence for high-altitude adaptation in Tibet. Science.

[136] Huerta-Sánchez E, Jin X, Asan, Bianba Z, Peter BM, Vinckenbosch N (2014). Altitude adaptation in Tibetans caused by introgression of Denisovan-like DNA. Nature.

[137] Bigham AW, Mao X, Mei R, Brutsaert T, Wilson MJ, Julian CG (2009). Identifying positive selection candidate loci for high-altitude adaptation in Andean populations. Hum Genomics.

[138] Scheinfeldt LB, Soi S, Thompson S, Ranciaro A, Woldemeskel D, Beggs W (2012). Genetic adaptation to high altitude in the Ethiopian highlands. Genome Biol.

[139] Alkorta-Aranburu G, Beall CM, Witonsky DB, Gebremedhin A, Pritchard JK, Di Rienzo A (2012). The genetic architecture of adaptations to high altitude in Ethiopia. PLoS Genet.

